# Supplementary material for: Frequency-related viscoelastic properties of the human incisor periodontal ligament under dynamic compressive loading
Source: PLoS One. 2020 Jul 13;15(7):e0235822. doi: 10.1371/journal.pone.0235822 (PMC7357742; doi:10.1371/journal.pone.0235822)
Supplement: S1 Data — (ZIP) [file pone.0235822.s001.zip › Cover letter .docx]

Department of Orthodontics

Affiliated Hospital of Stomatology

Nanjing Medical University

No.136 Hanzhong Road

Nanjing, 210029

P.R. China

Tel: 86-025-85031982

Fax:86-025-86516414

www.jsdental.cn

June 27, 2020

Dear Editor

Here we submit our revised version of original research article “*Frequency-related viscoelastic properties of human incisor periodontal ligament under dynamic compressive loading*” to *The PLOS ONE*.

The author list and affiliations are correct on the title page of our manuscript, and our author contributions are correct except Bin Wu are Conceptualization, Formal analysis, Methodology, Project administration, Visualization and Funding acquisition. There are some problems in financial disclosure and Funding Information, and we have corrected financial disclosure as listed below: This study was funded by Bin Yan, National Natural Science Foundation of China (Grant No.81571005), the Key Technology R&D Program of Jiangsu Province (BE2018723), and the Priority Academic Program Development of Jiangsu Higher Education Institutions (PAPD, 2018-87) and also funded by Bin Wu, National Natural Science Foundation of China (Grant No. 51305208), Jiangsu Provincial Key Laboratory of Oral Diseases Research Fund (JSKLOD-KF-1901). Funding Information was corrected as below:

National Natural Science Foundation of China	81571005	Dr. Bin Yan

the Key Technology R&D Program of Jiangsu Province	BE2018723	Dr. Bin Yan

Priority Academic Program Development of Jiangsu Higher Education Institutions (PAPD, 2018-87)	Dr. Bin Yan

National Natural Science Foundation of China	51305208	Dr. Bin Wu

Jiangsu Provincial Key Laboratory of Oral Diseases Research Fund (JSKLOD-KF-1901)	Dr. Bin Wu.

The funders had no role in study design, data collection and analysis, decision to publish, or preparation of the manuscript and the authors have no competing interests. Minimal data set is located in our manuscript and Supporting Information files, and our Data Availability Statement can be updated as follows: All relevant data are within the manuscript and its Supporting Information files.

We deeply appreciate your consideration of our manuscript. Please do not hesitate to contact me if you have any question or need further information about our manuscript.

Sincerely,

Bin Yan, DDS, PhD (Corresponding author)

Professor

Email: byan@njmu.edu.cn
