# Supplementary material for: Frequency-related viscoelastic properties of the human incisor periodontal ligament under dynamic compressive loading
Source: PLoS One. 2020 Jul 13;15(7):e0235822. doi: 10.1371/journal.pone.0235822 (PMC7357742; doi:10.1371/journal.pone.0235822)
Supplement: S1 Data — (ZIP) [file pone.0235822.s001.zip › Response to Reviewers.docx]

Response to Reviewers

We appreciate the reviewers’ positive comments and constructive suggestions on our manuscript, entitled “Frequency-related viscoelastic properties of the human incisor periodontal ligament under dynamic compressive loading” (PONE-D-19-34657). We have addressed all of the comments to the best of our ability, and we believe the revisions requested by the reviewers have significantly improved the quality of our manuscript. The reviewers’ unedited comments are provided below in **black** text followed by our responses in **blue** text.

1. If you would like to make changes to your financial disclosure, please include your updated statement in your cover letter.

**Authors’ reply:**

Thank you for your reminder. We have added another fund named Jiangsu Provincial Key Laboratory of Oral Diseases Research Fund (JSKLOD-KF-1901) which has also been stated it in our cover letter.

2. To enhance the reproducibility of your results, we recommend that if applicable you deposit your laboratory protocols in protocols.io, where a protocol can be assigned its own identifier (DOI) such that it can be cited independently in the future. For instructions see: <http://journals.plos.org/plosone/s/submission-guidelines#loc-laboratory-protocols>.

3. In your Methods section, please provide additional information regarding the human samples used in the study. Specifically:

- Please specify the source of the fresh corpses.

- State in your ethics statement whether the study involved the use of donated tissue/organs from any vulnerable populations.

- Provide information on the consent given by the donor or their next of kin. Examples of vulnerable populations include prisoners, subjects with reduced mental capacity due to illness or age, and children. If a vulnerable population was used, please describe the population and justify the decision to use tissue/organ donations from this group. If not, please state in your Ethics Statement, "None of the corpses were from a vulnerable population and all donors or next of kin provided written informed consent that was freely given."

- If the authors did not have access to any identifying information for the human samples, please state this.

**Authors’ reply:**

Thank you for your valuable review of our work. The fresh corpses came from Department of Anatomy of Nanjing Medical University with a declared legal consent given by the donor or their next of kin attained by the same administration. The study did not involve the use of donated tissue/organs from any vulnerable populations. We did not have access to any identifying information for the human samples.

More details have been provided in the revised paper, as listed below:

The fresh corpses used in this study were provided by the Department of Anatomy of Nanjing Medical University with a declared legal consent given by the donor or their next of kin attained by the same administration. The study did not involve the use of donated tissue/organs from any vulnerable populations. The authors did not have access to any identifying information for the human samples. (Details see page 6 line 119-123.)

3. We suggest you thoroughly copyedit your manuscript for language usage, spelling, and grammar. If you do not know anyone who can help you do this, you may wish to consider employing a professional scientific editing service.

Whilst you may use any professional scientific editing service of your choice, PLOS has partnered with both American Journal Experts (AJE) and Editage to provide discounted services to PLOS authors. Both organizations have experience helping authors meet PLOS guidelines and can provide language editing, translation, manuscript formatting, and figure formatting to ensure your manuscript meets our submission guidelines. To take advantage of our partnership with AJE, visit the AJE website (http://learn.aje.com/plos/) for a 15% discount off AJE services. To take advantage of our partnership with Editage, visit the Editage website (www.editage.com) and enter referral code PLOSEDIT for a 15% discount off Editage services. If the PLOS editorial team finds any language issues in text that either AJE or Editage has edited, the service provider will re-edit the text for free.

**Authors’ reply:**

Thank you for your valuable suggestion. We apologize the language issues in the previous manuscript. To make our paper easier to follow, the manuscript has been extensively reviewed and revised by American Journal Experts (AJE). We hope that the language in the revised paper has been substantially improved.

Reviewer #1	As a general comment regarding the way the results are presented, why Table 1 and Fig. 2 include the statistics of all the samples and then they are split into central and lateral incisors? Why not presenting then separately from the beginning? I couldn’t find an explanation of this fact in the text.

**Authors’ reply:**

Thank you for your valuable suggestion. Because the central incisor and the lateral incisor are adjacent teeth, and they are under similar bite force. Moreover, the central incisor and the lateral incisor are often retracted as a whole in orthodontic treatment. Therefore, we showed the results including the central incisor and the lateral incisor at the beginning to show the effect of frequency on the viscoelastic properties of the incisor PDL.

However, in a more detailed and specified view, the higher viscosity of the central incisor demonstrated that its PDL has a better energy-absorbing or shock-absorbing capacity for sudden loading than the lateral incisor, which indicates that the central incisor PDL can withstand more mastication force than the lateral incisor PDL. Since this particular comparison between the incisors in our study was of importance during orthodontic force application, we presented the central incisor and lateral incisor separately additionally to the scope of our study.

More details have been provided in the revised paper, as listed below:

In concordance with Tanaka [30], the higher viscosity of the central incisor in our study demonstrated that the central incisor PDL has a better energy-absorbing or shock-absorbing capacity for sudden loading than the lateral incisor, indicating that the central incisor PDL can withstand more bite force than the lateral incisor PDL. However, the central incisor and the lateral incisor are adjacent teeth and are often under similar bite force and retracted as a whole during orthodontic treatment (for details, see page 16, lines 311-317.)
